# Defining mammary basal cell transcriptional states using single-cell RNA-sequencing

**DOI:** 10.1038/s41598-022-08870-1

**Published:** 2022-03-22

**Authors:** Guadalupe Gutierrez, Peng Sun, Yingying Han, Xing Dai

**Affiliations:** 1 Department of Biological Chemistry, School of Medicine, University of California, D250 Med Sci I, Irvine, CA 92697-1700 USA; 2 The NSF-Simons Center for Multiscale Cell Fate Research, University of California, Irvine, CA 92697 USA

**Keywords:** Computational biology and bioinformatics, Developmental biology

## Abstract

Breast cancer is a heterogenous disease that can be classified into multiple subtypes including the most aggressive basal-like and triple-negative subtypes. Understanding the heterogeneity within the normal mammary basal epithelial cells holds the key to inform us about basal-like cancer cell differentiation dynamics as well as potential cells of origin. Although it is known that the mammary basal compartment contains small pools of stem cells that fuel normal tissue morphogenesis and regeneration, a comprehensive yet focused analysis of the transcriptional makeup of the basal cells is lacking. We used single-cell RNA-sequencing and multiplexed RNA *in-situ* hybridization to characterize mammary basal cell heterogeneity. We used bioinformatic and computational pipelines to characterize the molecular features as well as predict differentiation dynamics and cell–cell communications of the newly identified basal cell states. We used genetic cell labeling to map the in vivo fates of cells in one of these states. We identified four major distinct transcriptional states within the mammary basal cells that exhibit gene expression signatures suggestive of different functional activity and metabolic preference. Our in vivo labeling and ex vivo organoid culture data suggest that one of these states, marked by *Egr2* expression, represents a dynamic transcriptional state that all basal cells transit through during pubertal mammary morphogenesis. Our study provides a systematic approach to understanding the molecular heterogeneity of mammary basal cells and identifies previously unknown dynamics of basal cell transcriptional states.

## Introduction

The mammary gland contains an epithelial bilayer of basal and luminal cells that function in the production and secretion of milk from mother to offspring^1,2^. Multiple stem and progenitor cell populations have been identified in the basal and luminal layers, and the basal layer harbors multipotent stem cells that are capable of generating both basal and luminal progenies and reconstituting a functional mammary gland upon transplantation^3–5^. Distinct, small pools of stem cells in the basal layer have been identified by the expression of different markers such as *Procr*^6^, *Bcl11b*^7^*, Lgr5/Tspan8*^8^, *Cdh5*^9^, as well as by lineage-tracing *Axin2-*expressing cells^10^. However, basal cell heterogeneity in the normal mammary gland has been understudied despite knowledge of the existence of such stem cells. In particular, it is not clear how non-stem basal cells are transcriptionally organized or what their specific function and differentiation status might be.

Recent studies have utilized single-cell RNA-sequencing (scRNA-seq) to examine the normal mammary gland at single-cell resolution in both human^11^ and mouse^9,12,13^. These studies have provided foundational insights into the transcriptional landscape of the mammary epithelium. Other studies have also coupled transcriptional and epigenetic modalities at single-cell resolution to help uncover regulators of cellular identity within the mammary epithelium^13,14^. While these studies have advanced the understanding of the mammary epithelium, there still lacks a comprehensive and in-depth characterization of cellular states and differentiation landscape within the basal layer.

In this work, we aimed to characterize the transcriptional heterogeneity of mammary basal cells using scRNA-seq. Using differential gene expression analysis, we find four major basal cell transcriptional states. We provide a bioinformatic characterization of the molecular features of the cell states that suggest differential functional and metabolic activities. Using RNAScope, we validate the heterogeneous expression and differential enrichment of several scRNA-seq-identified basal transcriptional state markers, *Acta2*, *Tspan8*, and *Egr2*, in the intact mammary gland tissue. Lastly, we provide genetic cell labeling data to suggest that *Egr2*-expressing transcriptional state represents a dynamic one that all basal cells transit through during pubertal mammary morphogenesis. These findings regarding basal cell heterogeneity in the normal tissue lay the foundation for future work to probe the heterogeneity of their malignant counterparts in basal-like and triple negative breast cancer subtypes.

## Methods

### Mice

Wild-type C57BL/6 mice (Stock #000,664) and *ROSA26*^*mTmG*^ mice (Stock #007,576) were purchased from the Jackson Laboratory. *Egr2-Cre* mice were reported in a previous study^15^, and the following primers were used for genotyping:

Forward (*Egr2-Cre*): CGC TTC CTC GTG CTT TAC GGT AT (480-bp product);

Forward (WT): TCA TCA GTC GGG TTA GAG CTG (312-bp product);

Reverse: GGG CTG AGG AAG ACG ACT TTA.

*Egr2-Cre*;*ROSA26*^*mTmG*^ mice used for experimental analysis were generated by crossing *Egr2-Cre* males with *ROSA26*^*mTmG*^* females*. Crossing *Egr2-Cre;ROSA26*^*mTmG*^ males with WT females resulted in 100% GFP expression in the mammary epithelium of female *Egr2-Cre;ROSA26*^*mTmG*^ offspring (data not shown), suggesting germline expression of *Egr2*. Mice were maintained in a pathogen-free facility, following procedures that conform and have been approved by the University of California Irvine Institutional Animal Care and Use Committee.

### Flow cytometry

Flow cytometry was performed as described^16,17^. Briefly, mammary glands were isolated, minced with a razor blade, and incubated with 300 U/mL collagenase (Sigma, C9891) and 100 U/mL hyaluronidase (Sigma, H3506) for 90 min at 37 °C. The cells were treated with red blood cell lysis buffer (Sigma, R7757) for 5 min at room temperature before treating with 0.25% Trypsin (Gibco, 25,200) for 2 min at 37 °C and dispase II (2 mg/mL) (Stem Cell Technologies, 07,913) with 0.1 mg/mL DNase I (Sigma, DN25) for 2 min at 37 °C. Cells were stained for 30 min in the dark at room temperature using APC-CD45 (BD Biosciences, 559,864; 1:250), APC-CD31 (BD Biosciences, 551,262; 1:250), APC-Ter119 (BD Biosciences, 557,909; 1:250), PE/C7-Epcam (Bio Legend, 118,215; 1:250), and PerCP-Cy5.5-Cd49f (Bio Legend, 555,735; 1:250). Cells were washed and stained with Sytox blue (Invitrogen, S34857; 1:1000) before flow cytometry.

### scRNA-seq analysis

scRNA-seq experiments were performed in two separate runs using the 10X Genomics platform on FACS-sorted mammary epithelial cells from 8–9-week-old virgin females. Experimental details were as described in another study^18^. The sequencing data were analyzed using R version 3.6.1 and Seurat 3.1.0. Low quality cells were filtered based on mitochondrial DNA content, total number of transcripts, and total number of genes detected. Basal cells (*Krt14*^+^ cluster) were computationally isolated for further analysis. STRING version 11.0 was used to examine the protein–protein interactions using the marker genes identified by differential expression test using Seurat. Disconnected nodes were removed from the graphs, and the default interaction score (medium confidence; 0.4) was used to identify interactions. RNA Velocity analyses were performed using the R package velocyto.R (linear model) and nlvelo (non-linear model). The R package CellChat was used for analyzing the ligand-receptor communications of cells.

### RNAScope

RNAScope was performed as previously described^19^ using ACD Bio’s reagents. Briefly, #4 mammary glands from mice were frozen in OCT and cryosectioned at 10 µm. Sectioned tissues were fixed for 1 h at room temperature with 4% PFA, and the RNAScope Multiplex Fluorescent v2 assay was run per manufacturer’s recommendations using the following probes: *Acta2* (319,531-C3), *Tspan8* (842,941-C1), and *Egr2* (497,871-C2). Images were obtained using a Zeiss LSM700 confocal microscope and quantified using FIJI software.

### Whole-mount immunofluorescence imaging

Mammary glands (#4) of 3-week-old *Egr2-Cre;ROSA26*^*mTmG*^ and *ROSA26*^*mTmG*^ mice were surgically isolated from adjacent tissues and spread on glass slides. Images were acquired using a Keyence BZ-X710 microscope (Keyence Corporation, Itasca, Illinois, USA).

### Ethics and approval and consent to participate

All mouse experiments have been approved by and conform to the regulatory guidelines of the Institutional Animal Care and Use Committee of the University of California, Irvine. The study is reported in accordance with ARRIVE guidelines.

## Results

### Identification of four basal cell transcriptional states in adult virgin mouse mammary gland

**Figure 1:**
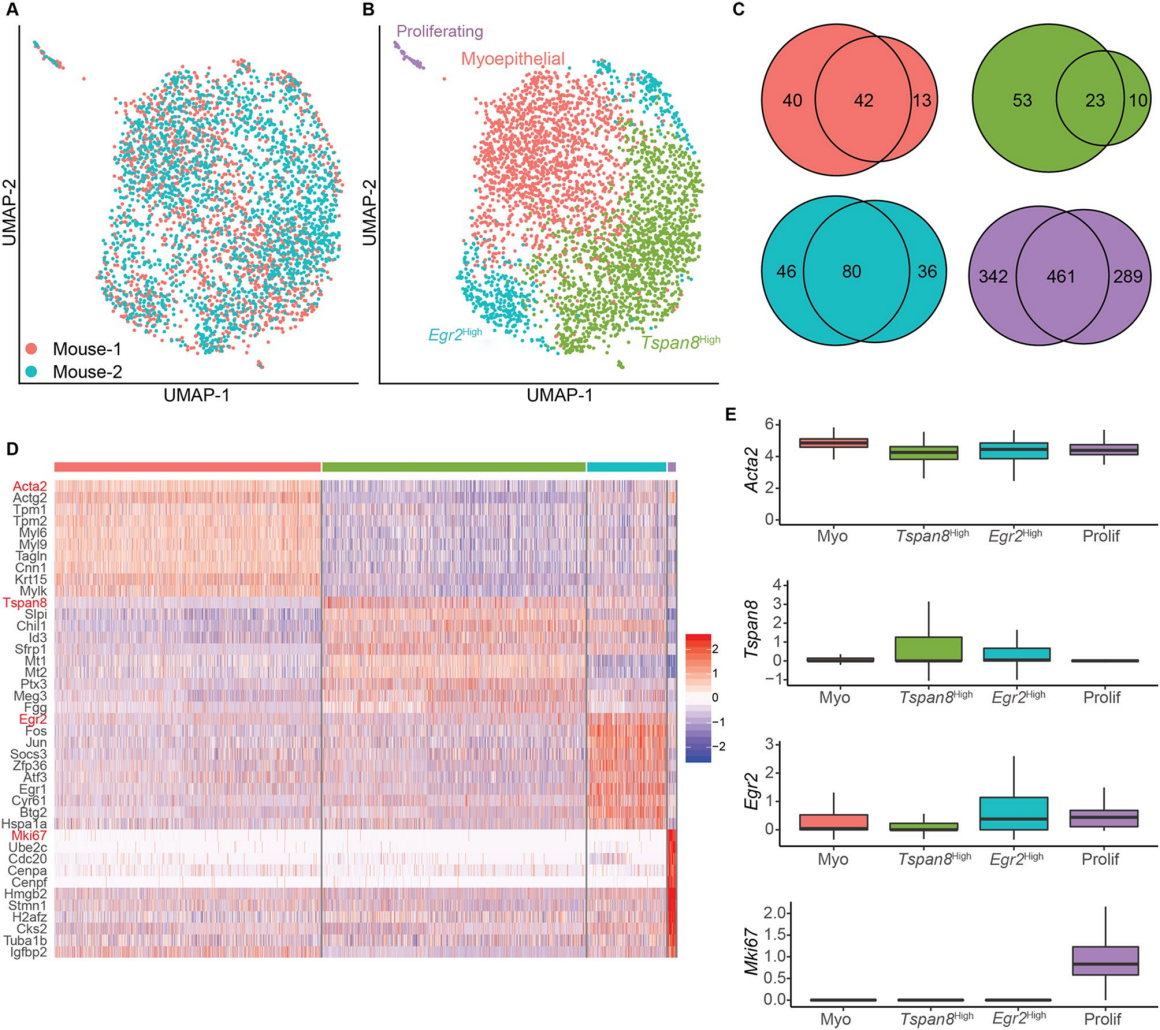
Identification of four major transcriptional states in basal cells of adult virgin mammary gland. (**A**) UMAP projection of mammary basal cells colored by mouse. (**B**) UMAP projection as in (**A**) colored by transcriptional state. (**C**) Venn diagram showing overlap in marker genes for the transcriptional states identified in both mice. Each circle represents the number of marker genes for a particular transcriptional state [same color code as in (**B**)] in each mouse. (**D**) Heatmap of top 10 marker genes for each transcriptional state identified in (**B**). (**E**) Boxplots of the indicated marker genes (marked in red in **D**) across all four basal transcriptional states.

Previously, we performed scRNA-seq analysis on fluorescence-activated cell sorting (FACS)-isolated mammary epithelial cells (including both basal and luminal populations) from 8–9-week-old virgin females to characterize the heterogeneous expression of epithelial-to-mesenchymal transition (EMT)-associated genes^18^. However, a systematic characterization of the transcriptional diversity of basal cells has not been done in that study. To achieve this, we computationally subset the 3,651 cells that express basal cell marker *Krt14* from the dataset for further analysis. Visualizing these basal cells in a UMAP projection suggests that there are no obvious batch effects (Fig. 1A). Clustering and differential gene expression analyses revealed the presence of four clusters: (1) a cluster enriched for classical myoepithelial genes (*Acta2, Actg2)* and thus termed “myoepithelial”; (2) a *Tspan8*^High^ cluster enriched for genes the high expression of which has been previously shown to identify stem cells in the mammary basal compartment (*e.g. Tspan8*^8^, *Epcam*^20^); (3) an *Egr2*^High^ cluster enriched for early response- and stress-related genes (e.g. *Egr2*, *Fos*, *Jun*)^21^; and (4) a small cluster marked by proliferation-associated genes (e.g., *Mki67*, *Pcna*) (Fig. 1B–D; Supplemental Table 1). We also observed the same four cell states when each mouse was analyzed individually (Fig. S1A–D), and the marker genes used to identify each cell state in the mice showed a high degree of overlap (Fig. 1C). Importantly, the marker genes that discriminated the different transcriptional states were differentially expressed but not mutually exclusive (Fig. 1E).

To examine the robustness of these transcriptional states, we also sequenced FACS-sorted mammary epithelial cells from 8–9-week-old virgin mice deficient in EMT-inducing transcription factor *Zeb1*^18^ and computationally subset basal cells for further analysis (Fig. S1E, F). In addition to a proliferating basal cell population, three other basal transcriptional states were observed in each mutant mouse analyzed (Fig. S1G–J). The top markers of each of these transcriptional states were largely similar, albeit not identical, to those in the wild-type mice. Therefore, even in *Zeb1*-deficient mammary glands where basal stem cell function is compromised^18^, similar molecular heterogeneity of bulk basal cells still exists, featuring four major transcriptional states.

We also interrogated the published scRNA-seq data on *KRT14*-expressing cells of the human breast from three individuals^11^. Generally, top marker genes for the three “non-proliferating” mouse basal cell states were detected at much lower frequencies in these human samples (Supplemental Table 2). For example, *Acta2*, which has been previously reported to be expressed in > 96% of mouse mammary basal cells^20^, was detected in 99.6% of the mouse basal cells in our scRNA-seq analysis but *ACTA2* was only detected in 38.6% of the human *KRT14*^+^ cells. Moreover, *Egr2* was expressed in 29.8% of mouse basal cells, but *EGR2* was only detected in 5.6% of human *KRT14*^+^ cells. Nevertheless, we were still able to observe transcriptional states in the human *KRT14*^+^ population that resembled the “myoepithelial” and proliferating basal cell states in mouse (Fig. S1K–M). These data reveal both disparities and similarities in the transcriptional states of mammary basal cells between mouse and human.

### Molecular features of the basal cell states

**Figure 2:**
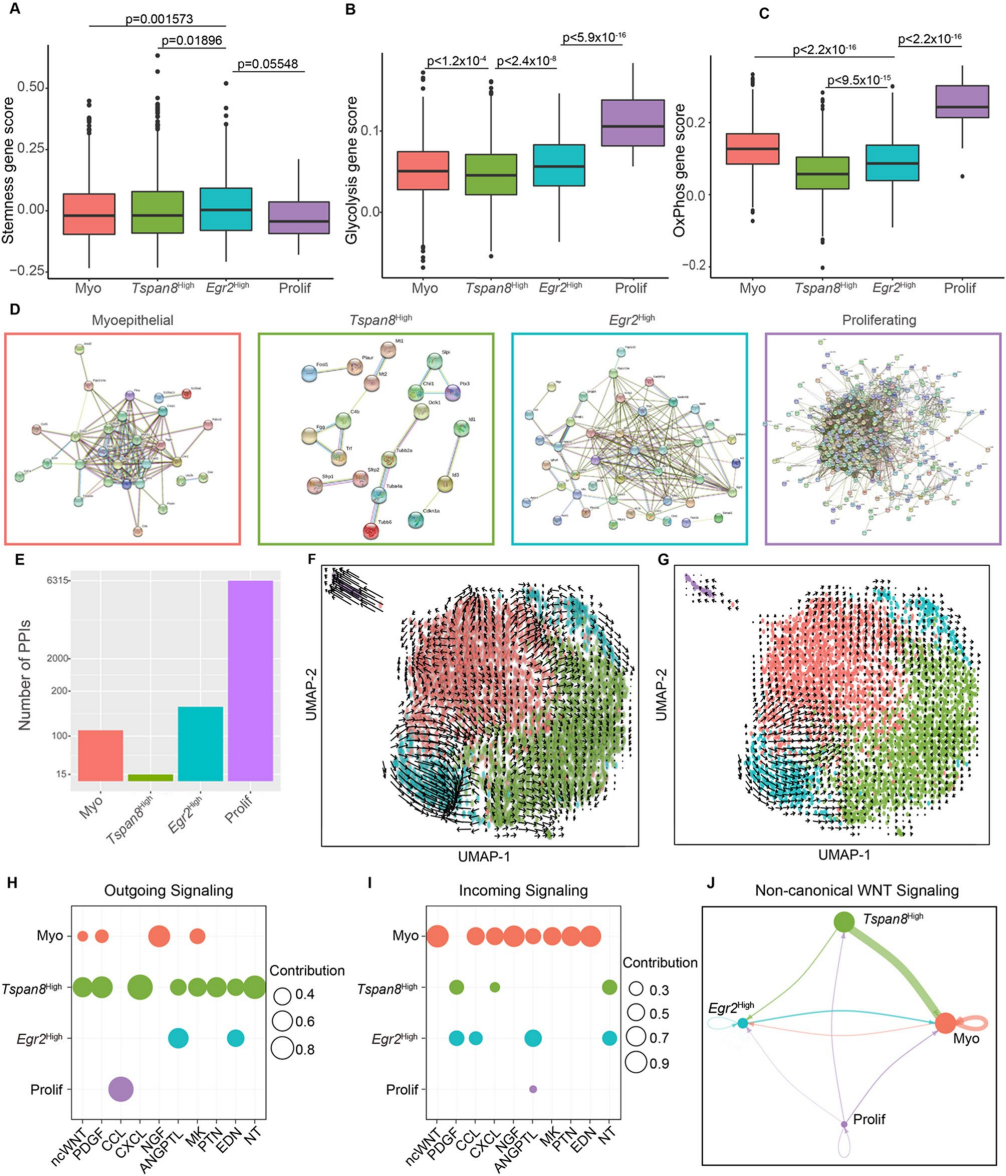
Molecular features of the basal transcriptional states. (**A**–**C**) Boxplots displaying gene scoring of each basal transcriptional state using gene signatures for mammary stemness (**A**), glycolysis (**B**), and OxPhos (**C**). *p* values in (**A**-**C**) were generated using Mann–Whitney *U* tests. (**D**) PPIs for each cell state (myoepithelial, pink; *Tspan8*^High^, green; *Egr2*^High^, blue; proliferating, purple), where each node is protein coded by the marker gene and each edge is a predicted direct or indirect interaction. (**E**) Bar plots displaying the number of PPIs for each transcriptional state. (**F**–**G**) Projections of RNA Velocity fields onto UMAP from Fig. 1B using linear (**F**) and non-linear (**G**) models. (**H**–**I**) Dot plots indicating the outgoing (**H**) and incoming (**I**) signaling contributions from each transcriptional state for significant signaling pathways identified. (**J**) Signaling pattern of non-canonical WNT signaling.

Next, we sought to characterize the molecular features of the basal cell states in mice. Intrigued by their upregulated expression of known stemness-associated genes *Tspan8* and *Epcam*, we wondered if *Tspan8*^High^ cells also display enriched expression of other genes reported to mark mammary basal/stem cells (*e.g. Procr*^6^, *Bcl11b*^7^, *Cdh5*^9^, *Lgr5*^8^). Using a gene scoring approach to calculate the average expression of a “stemness gene” signature (Supplementary Table 3), we found that the *Egr2*^High^ basal cells had the highest average score whereas the proliferating basal cells the lowest (Fig. 2A). Overall, the discriminating power of this signature is non-remarkable.

Differential metabolic preference of mammary basal and luminal cells has been suggested, such that basal cells may prefer a glycolytic metabolism while luminal cells display increased oxidative phosphorylation^22,23^. This metabolic paradigm is of interest, given that cancerous cells have increased glycolytic activity^24^. Using a set of hallmark gene sets defined by the Molecular Signatures Database to score for glycolysis and oxidative phosphorylation (OxPhos) (Supplementary Table 3), we found that the proliferating basal cells have the highest glycolytic and OxPhos signatures (Fig. 2B,C), suggesting increased requirement for energy to support cellular growth and division. Of the “non-proliferating” basal cell states, the *Egr2*^High^ cells scored the highest for the glycolytic signature, and the “myoepithelial” cells scored the highest for OxPhos. Interestingly, the *Tspan8*^High^ cells scored the lowest for both signatures. These data reveal previously unrecognized metabolic heterogeneity within the mammary basal layer.

To gain further insights into the potential activity of each basal transcriptional state, we examined the protein–protein interaction (PPI) networks of all of the marker genes that define each state. We utilized STRING^25^ to derive direct (physical) and indirect (functional) associations between the marker genes. Interestingly, the number of PPIs were vastly different across the cell states, with proliferating basal cells exhibiting the highest number of PPIs and the highest number of marker genes (Fig. 2D,E; Fig. S2A), which is consistent with their highly specialized cellular activity. Although the difference in the number of marker genes for each “non-proliferating” cell state was small, the difference in the number of PPIs was more pronounced (Fig. 2E; Fig. S2A). For example, the numbers of PPIs in the *Tspan8*^High^ and “myoepithelial” cells were 15 (lowest of all four states) and 113 (second highest), respectively, whereas their numbers of unique marker genes were 31 (lowest) and 39 (second lowest). This data suggest that the marker genes used to define the “myoepithelial” cells have a higher degree of physical and functional associations and may point to a functional consequence. Consistently, a gene ontology (GO) term analysis using Enrichr^26,27^ to probe the GO Biological Processes 2018 library revealed terms related to muscle contraction for the “myoepithelial” marker genes (Fig. S2B).

Next, we performed RNA Velocity^28^, a computational method that calculates the relative abundances of spliced and unspliced RNA to infer the future states of single cells. Based on the directions of the vectors arrows, which are known to associate with possible state transitions, we did not observe a clear differentiation trajectory across the different basal cell states regardless of whether we used a linear^28^ (Fig. 2F) or non-linear model^19^ (Fig. 2G) of RNA Velocity. However, we found consistent differences in the length of the vector arrows that suggest differential RNA dynamics among the different states. The “myoepithelial” and *Tspan8*^High^ cells showed small RNA velocities (short or no arrows), known to associate with either quiescent or terminally differentiated cells^29,30^. The *Egr2*^High^ cells exhibited large RNA velocities in both linear and non-linear models (Fig. 2F,G), suggesting that these cells may be in a more active and transitional cellular state compared to the others. The proliferating cells exhibited large RNA velocities in the linear but not in the perhaps more realistic non-linear model^19^, and in both cases, the arrows pointed away from but not back to the “non-proliferating” cells (Fig. 2F,G), raising the possibility that they may not be able to readily switch back to a “non-proliferating” state^19^.

Lastly, we used CellChat^31^ to explore ligand-receptor pairs and infer potential signaling cross-talks within the basal cell layer. CellChat identified ten signaling pathways that were significantly enriched within basal cells, and outgoing and incoming signals were largely heterogeneous across the cell states (Fig. 2H,I). It appeared that the *Tspan8*^High^ cells send the most outgoing signals, and the “myoepithelial” cells receive the most incoming signals. Interestingly, non-canonical WNT (ncWNT) signaling surfaced as the most prominent in “myoepithelial” cells, which appeared to be the primary cells responding to WNT signals from the *Tspan8*^High^ cells (i.e., paracrine) and, to a lesser extent, from the “myoepithelial” cells themselves (i.e., possibly autocrine) (Fig. 2J).

Collectively, our findings suggest that the *Tspan8*^High^ transcriptional state associates with low number of marker genes, low PPIs, low glycolysis, slow RNA dynamics, but can potentially serve as a signaling niche^32^, whereas the *Egr2*^High^ transcriptional state is the most dynamic of all states and the “myoepithelial” transcriptional state represents the most specialized state associated with a “mature” myoepithelial fate.

### Validating basal transcriptional heterogeneity and detecting dynamic *Egr2* expression in the intact mammary tissue

**Figure 3:**
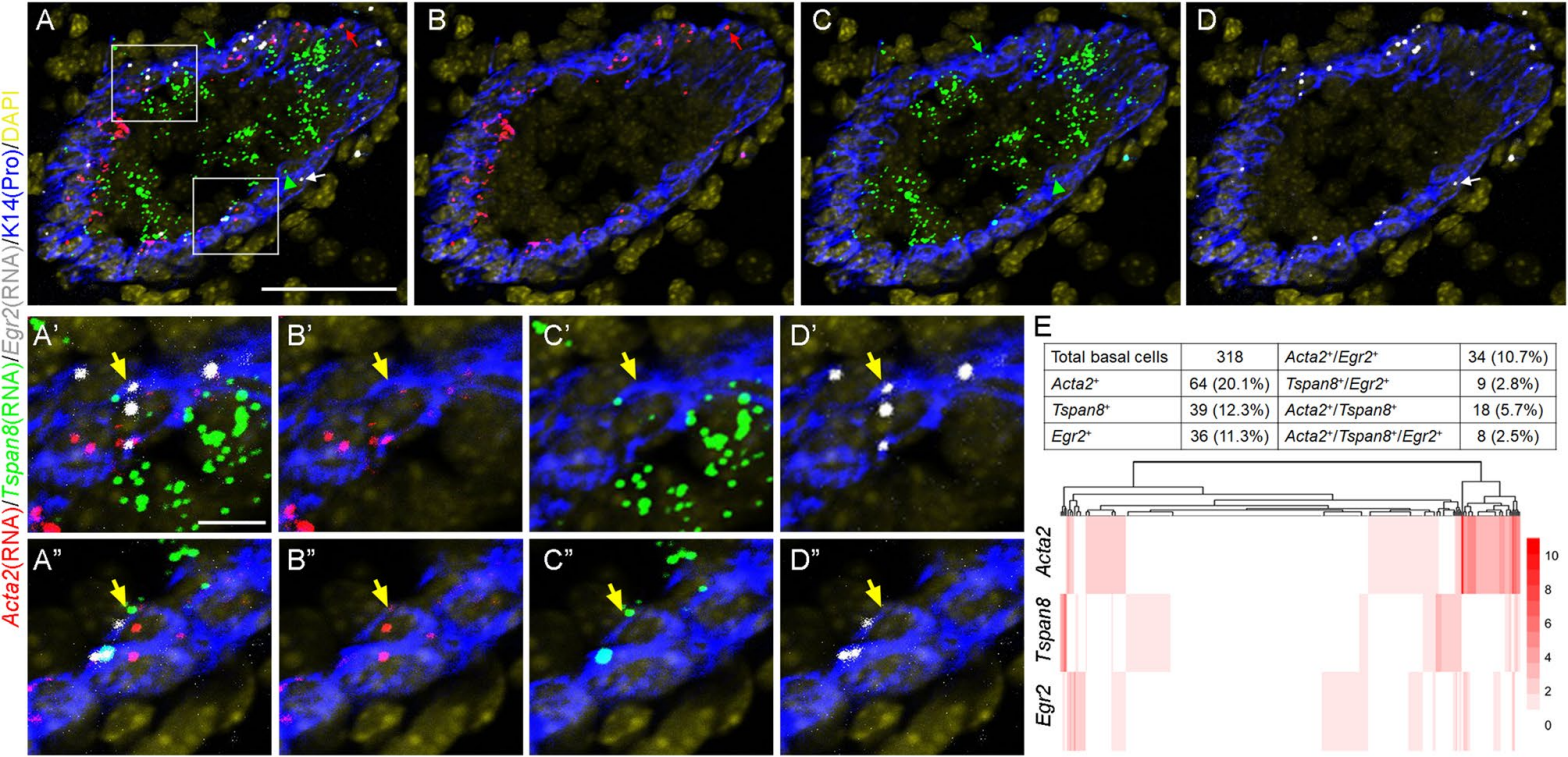
RNAScope validation of differential expression of several basal transcriptional state markers. (**A**–**D**) Representative 5-channel (**A**) and 3-channel (**B**–**D**) images showing the expression of *Acta2* (red), *Tspan8* (green), *Egr2* (white) in the mammary gland of 10-week-old virgin females. K14 protein immunostaining (blue in all images) marks the basal cells. DAPI stains the nuclei (artificially colored yellow in all images). Red, green, and white arrows indicate a basal cell with only *Acta2*, *Tspan8,* and *Egr2* expression, respectively. Green arrowhead indicates a *Tspan8-*expressing K14-positive cell that resides in basal-luminal border. Boxed areas in (**A**) are enlarged in (**A**ʹ–**D**ʹ) and (**A**ʺ–**D**ʺ) to highlight basal cells with more than one marker detected (indicated by yellow arrow). Scale bar = 50 μm in (**A**–**D**); 10 μm in (**A**ʹ–**D**ʹ) and (**A**ʺ–**D**ʺ). (**E**) Quantification results for experiments as in (A-D). Table summarizing the numbers of single, double, and triple-positive cells is shown at the top, and heatmap with hierarchical clustering using the quantified expression of probes shown at the bottom.

We next sought to examine the molecular heterogeneity in bulk basal cells in the intact mammary tissue by using RNAScope to validate the differential expression of a top marker gene from each “non-proliferating” basal transcriptional state. Specifically, we probed for the expression of *Acta2*, *Tspan8*, and *Egr2* mRNAs simultaneously in mammary gland of adult virgin mice. Indeed, expression of each transcriptional state marker gene was detected in only a subset of basal cells (Fig. 3A–E; Fig. S3A–H). There did not appear to be any notable spatial patterning of these transcriptional state markers, except that a small number of *Tspan8*^+^ cells that are positive for K14 (indicating a basal cell fate^2,33^) seem to occupy positions between basal and luminal layers (Fig. 3A-D; Fig. S3A–H). Consistent with scRNA-seq data, *Acta2* showed the highest coverage in basal cells (~ 39%) (Fig. 3E). This is less than the 99% detection rate in our scRNA-seq data, likely reflecting differential sensitivity of different detection methods. *Tspan8* transcripts were detected in ~ 23% of the basal cells (Fig. 3E), and its most abundant expression was actually seen in the luminal layer, a finding confirmed by our scRNA-seq data (Fig. S3I). *Egr2* mRNAs were detected in ~ 27% of the basal cells (Fig. 3E) and expression was barely detectable in luminal cells, and this basal-restricted pattern was also seen in our scRNA-seq data (Fig. S3J). Hierarchically clustering basal cells by their expression (number of RNAScope signal foci/per nucleus) of the marker genes revealed groups of single basal cells that expressed a single marker gene (Fig. 3E). Overall, only a small fraction of basal cells (~ 3%) expressed all three markers, and a sizable fraction (~ 35%) of cells did not exhibit detectable expression of any of the makers examined (Fig. 3A′–D′,A″–D″,E). Together, these data uncover the scRNA-seq-predicted transcriptional heterogeneity in the basal compartment of the intact mammary tissue.

**Figure 4:**
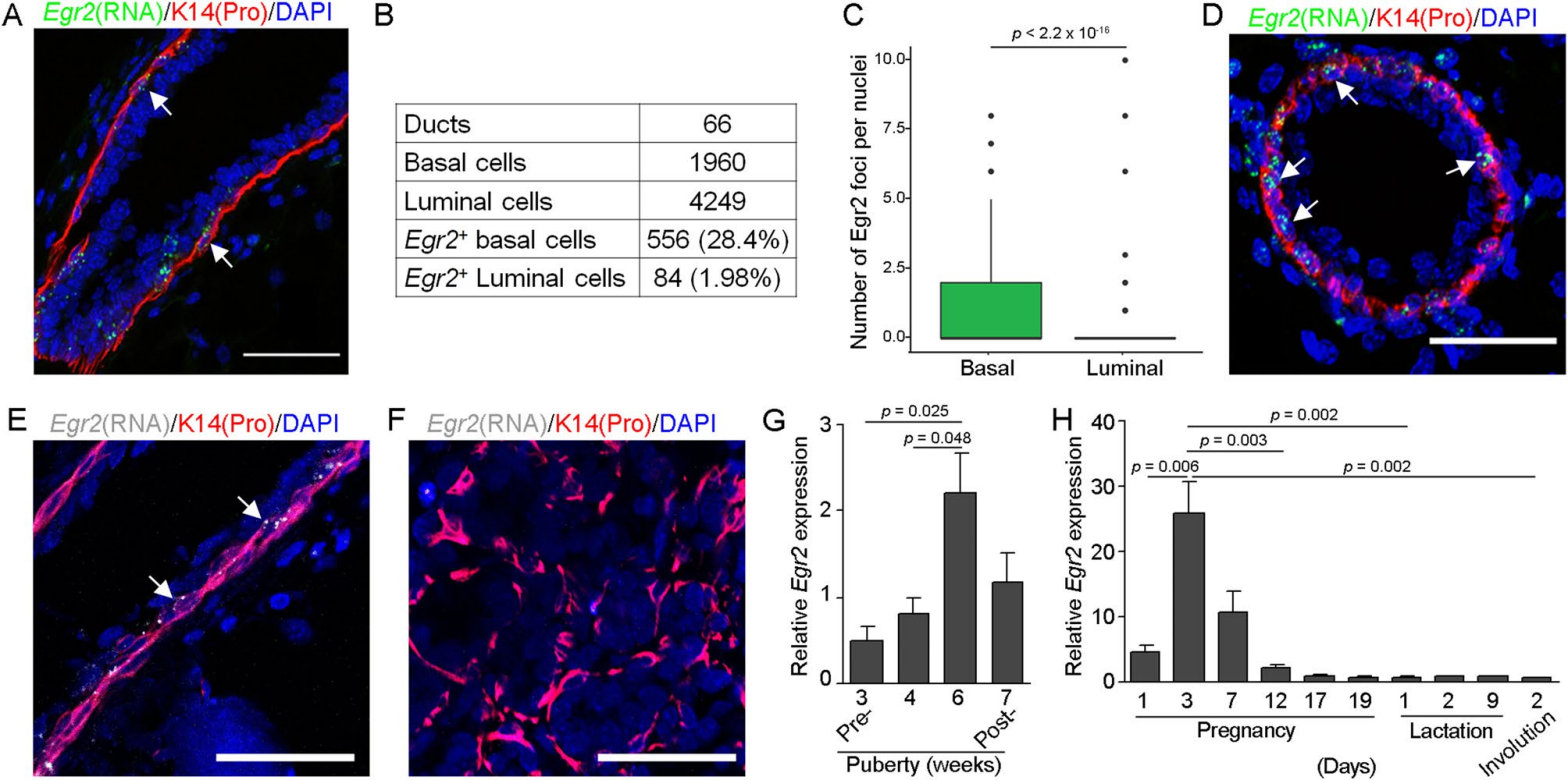
Dynamic *Egr2* mRNA expression in mammary gland. (**A**–**D**) RNAScope detection of *Egr2* expression (green) in mammary gland from 10-week-old virgin mice. K14 antibody (red) stains the base cells and DAPI (blue) stains the nuclei. Representative image of a single mammary duct is shown in (**A**), and result of quantitative analysis (using multiple ducts as indicated) of the frequency of *Egr2-*expressing basal or luminal cells is shown in (**B**). (**C**) Boxplots displaying the number of *Egr2* mRNA (green) foci per cell in basal or luminal cells. (**D**–**F**) RNAScope detection of *Egr2* mRNA (white) in mammary gland from 3-day (**D**) or 15-day (**E**, **F**) pregnant mice. Scale bar = 50 μm in (**A**), (**D**–**F**). White arrows indicate positive *Egr2* signal. (**G**–**H**) Bar plots indicating the expression of *Egr2* in whole mammary gland across the reproductive cycle using previously published data (GEO accession # for G: GSE6453; accession # for H: GSE8191). *p* values in (**C**) and (**G**–**H**) were generated using a Student’s *t*-test.

Expression of early-response genes such as *Egr2* has been reported as an artifact of stresses induced by the tissue dissociation/sequencing protocol^21^, but our detection of *Egr2*-expressing basal cells in the mammary tissue suggests the physiological existence of an *Egr2*^High^ basal transcriptional state. To probe this further, we performed RNAScope on mammary gland of different reproductive stages using the *Egr2* probe alone. In adult virgin gland, a significant enrichment of *Egr2* foci was again observed in the basal layer relative to the luminal layer, with *Egr2* mRNA being detected in ~ 28% of basal cells but in only ~ 2% of luminal cells (Fig. 4A–C). Basal-enriched *Egr2* expression was also abundantly detected in the mammary ducts of pregnant mice at day 3 of pregnancy (Fig. 4D). By day 15 of pregnancy, *Egr2*-expressing cells became less frequent in the ductal basal compartment, and were barely detectable in the alveolar basal compartment (Fig. 4E,F). We also probed the expression of *Egr2* using previously published microarray data on whole mammary gland across different stages of the reproductive cycle^34^, which revealed significantly upregulated *Egr2* expression during puberty (5–6 weeks of age) and early pregnancy (day 3) but very low expression during late pregnancy (day 17–19), lactation, and involution (Fig. 4G,H). Taken together, these data demonstrate physiologic and dynamic expression of *Egr2* in the mammary gland that coincides with periods of active epithelial morphogenesis and ductal expansion.

### Genetic evidence for basal-biased expansion of *Egr2*-expressing cells during pubertal mammary gland development

**Figure 5:**
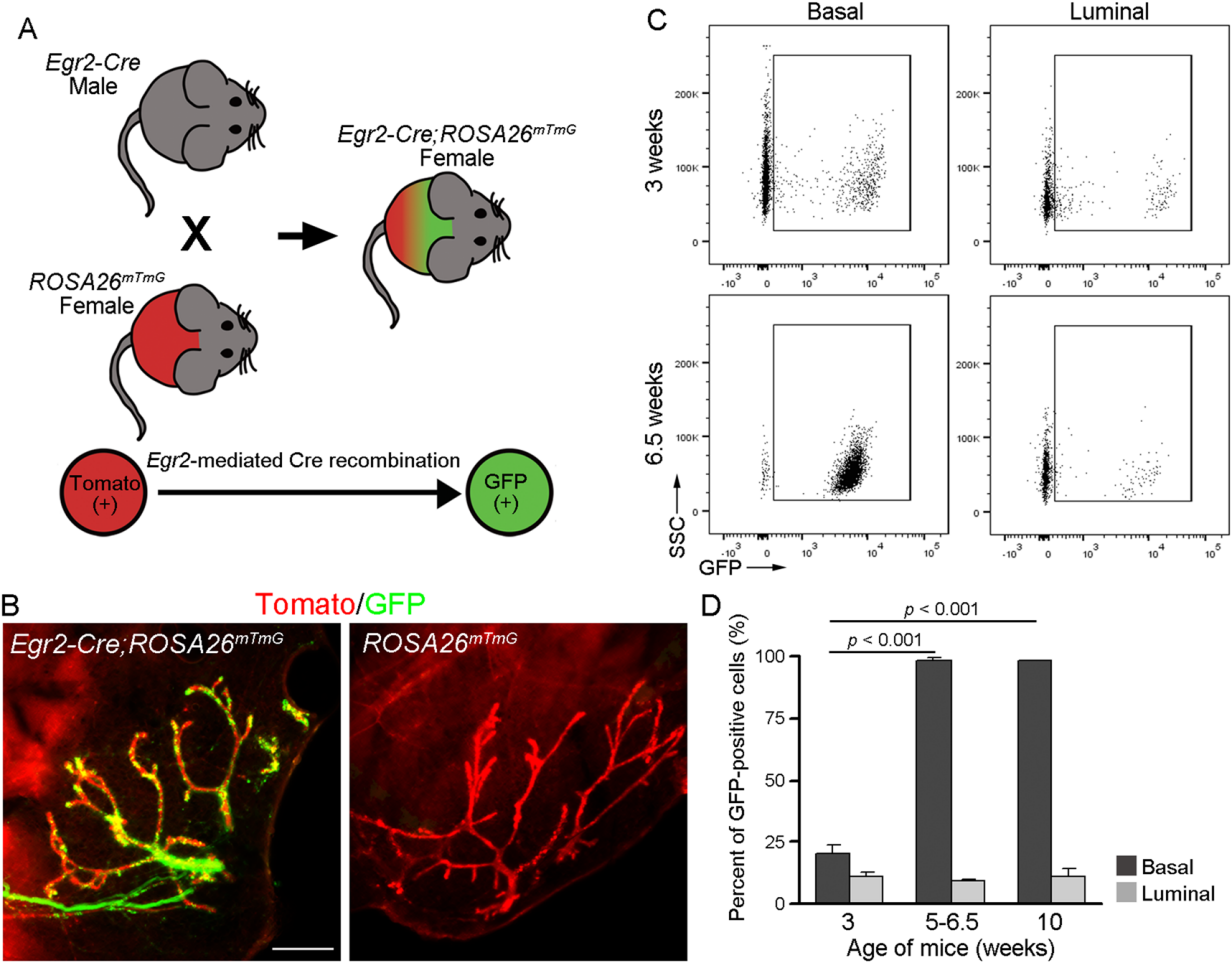
Genetic labeling of *Egr2*-expressing cells and progenies in mammary gland. (**A**) Experimental design. (**B**) Whole-mount imaging of GFP and tdTomato fluorescence in mammary gland from 3-week-old *Egr2-Cre;ROSA26*^*mTmG*^ or *ROSA26*^*mTmG*^ mice. Scale bar = 500 μm. (**C**, **D**) Representative flow profiles (**C**) and summary bar plots (**D**) displaying the number of GFP-positive cells in basal and luminal cells during pubertal mammary development. *p* value was generated using a Student’s *t*-test. n = 3 *Egr2-Cre;ROSA26*^*mTmG*^ mice per age analyzed.

*Egr2* is of interest because its expression marks a population of actively expanding hair follicle progenitor cells^15^ and it encodes a transcription factor that regulates the expression of *Notch1*, a critical gene involved in mammary basal-luminal binary differentiation^35^. To track *Egr2*-expressing cells in vivo, we crossed *Egr2-Cre* (*Krox20-Cre*) mice^15^ with *ROSA26*^*mTmG*^ reporter mice to generate *Egr2-Cre*; *ROSA26*^*mTmG*^ females, where *Egr2*-expressing cells and their progenies are fluorescently marked by GFP expression (Fig. 5A). To visualize the spatial location of *Egr2*-expressing cells and progenies, we performed whole-mount imaging analysis of GFP and tdTomato fluorescence in mammary gland of 3-week-old *Egr2-Cre*; *ROSA26*^*mTmG*^ mice. While no GFP^+^ cells were detected in the *ROSA26*^*mTmG*^ control mice as expected, such cells were found throughout the rudimentary ducts as well as in terminal end buds of *Egr2-Cre*; *ROSA26*^*mTmG*^ mice (Fig. 5B).

Flow cytometry analysis using GFP in conjunction with cell lineage surface markers (e.g., Cd49f, Epcam) revealed that 15–27% of the basal cells in mammary gland from 3-week-old (pre-puberty) *Egr2-Cre*; *ROSA26*^*mTmG*^ females were GFP^+^ (Fig. 5C,D). By mid-puberty (6.5 weeks of age) and in adulthood (10 weeks of age), the number of GFP^+^ basal cells dramatically increased to near 100% of all basal cells (Fig. 5C,D). The surface expression levels of Cd49f and Epcam in GFP^+^ and GFP^-^ basal cells were similar (Fig. S4A). At all ages examined, less than 10% of the luminal cells showed GFP expression (Fig. 5C,D). Taken together with the scRNA-seq and RNAScope findings above that fewer than a third of the basal cells in adult virgin gland showed detectable *Egr2* mRNA expression, these flow data suggest that virtually all basal cells in the adult gland had transited through an *Egr2*-expressing state at some point during pubertal mammary gland development and/or are derived from *Egr2*-expressing basal cells. Further illustrating the dynamic nature of *Egr2* expression in basal cells, FACS-sorted GFP^-^ basal cells from mammary glands of 3-week-old *Egr2-Cre*; *ROSA26*^*mTmG*^ females were able to activate GFP expression de novo under organoid culture conditions, whereas GFP^+^ basal cells remained positive over multiple passages (Fig. S4B).

## Discussion

To date, several scRNA-seq studies have been conducted on human and mouse mammary glands, each presenting a unique perspective on epithelial cellular composition, differentiation dynamics, and stem cell prediction. Previous studies have also delved into basal cell heterogeneity and the presence of rare stem cells in the basal layer^6,7,9,10,36–38^, but the gene expression program that underlies bulk basal cell dynamics and differentiation has not been clearly elucidated. Our study adds to the list of existing datasets and provides a deeper and comprehensive analysis of the transcriptional heterogeneity within mammary basal cells. Our analysis shows that bulk basal cells exist in at least four distinct transcriptional states that can be identified by their unique enrichment for the expression of specific gene signatures associated with potential stemness (e.g., *Tspan8*^high^ state), differentiation status (e.g., mature myoepithelial state), and/or rapid cellular dynamics and responses (e.g., *Egr2*^High^ and proliferating states). Intriguingly, our data does not point to a unidirectional, hierarchical differentiation trajectory originating from one state and ending in another. Instead, they suggest that mammary basal cells adopt dynamic non-hierarchical transcriptional states, with the exception that the proliferating state may not readily revert back to any of the “non-proliferating” state. This dynamics implies the inherent plasticity of any given basal cell, a notion that is consistent with the demonstrated functional plasticity of adult basal cells especially upon transplantation, and that mature myoepithelial cells possess regenerative stem cell activity that can manifest under appropriate conditions^4,5,10,20^. While it may be technically challenging to sort and purify the basal cell subsets in different transcriptional states due to overlapping expression of cell surface markers, future experiments to generate and analyze Cre-expressing alleles driven by temporally controlled promoters of genes encoding select basal transcriptional state markers will enable lineage tracing of the fates and activities of basal cells in each state during mammary development, regeneration, and tumorigenesis.

We were able to confirm the expression of several basal transcriptional state markers in the intact mammary gland using in situ mRNA detection and found them to be largely non-overlapping albeit not mutually exclusive. It has been reported that nearly all basal cells in the mammary gland, including stem cells, express *Acta2* at the mRNA and protein levels^20^. Our scRNA-seq data support this notion by revealing that > 99% of basal cells show detectable expression of *Acta2.* This said, we found that a fraction of them show heightened expression of *Acta2* and other myoepithelial-related genes; these are likely the same cells in which *Acta2* expression was over the detection threshold of RNAScope analysis and they may represent mature myoepithelial cells. The *Egr2*^High^ transcriptional state is of particular interest because of its faster cellular dynamics relative to the other states. Our RNAScope experiments detected the mRNA expression of *Egr2* in a subset of basal cells in the intact tissue. Moreover, in scRNA-seq, single-probe, and multi-probe RNAScope experiments, we observed remarkable consistency in the precise frequency (27–30%) of *Egr2*-positive cells, indicating robustness of the observation.

In the hair follicle, another leading model of adult stem cell biology, *Egr2* expression marks the matrix cells, which are highly proliferative but transit-amplifying progenitor cells that terminally differentiate to produce the hair shaft^15^. In the mammary gland, such transient amplifying progenitor cells remain elusive. Our proof-of principle data based on *Egr2-Cre*-mediated GFP fluorescence is consistent with the possibility that *Egr2-*expressing mammary basal cells are such progenitor cells and serve as the workhorse during pubertal mammary gland development to generate nearly the entire basal cell compartment of mature gland. However, an alternative, and perhaps more likely based on our RNA Velocity and organoid culture data, possibility is that *Egr2*^High^ is simply a transcriptional state that all basal cells transit through at some point during pubertal mammary development. It will be worthwhile to generate *Egr2-Cre*ER mice in the future to seek definitive evidence for the function and fate of the *Egr2*^+^ basal cell subset during mammary gland development, regeneration, and tumorigenesis.

Overall, our study provides a systematic analysis of mammary basal cell heterogeneity and a useful reference for future investigation into how basal cell gene expression changes during breast cancer initiation and progression. A thorough understanding of the transcriptional heterogeneity of normal mammary basal cells and their malignant counterparts might be particularly relevant in the development of differentiation therapies for basal-like and triple negative breast cancers.

## Conclusions

Our results have identified four major transcriptional states within the mammary basal cells that exhibit gene expression signatures suggestive of different functional activity and metabolic preference. Our in vivo data suggest that one of these transcriptional states, marked by *Egr2* mRNA expression, represents an actively expanding and/or obligatory transitional state during pubertal mammary morphogenesis. These findings regarding basal cell heterogeneity in the normal tissue lay the foundation for future work to probe the heterogeneity of the malignant counterparts in basal-like and triple negative breast cancer subtypes.

## Supplementary Information

Supplementary Figure S1.

Supplementary Figure S2.

Supplementary Figure S3.

Supplementary Figure S4.

Supplementary Table 1.

Supplementary Table 2.

Supplementary Table 3.

Supplementary Legends.

## Data Availability

The single-cell datasets used and analyzed during the current study are available under Accession #GSE155636 (https://www.ncbi.nlm.nih.gov/gds). Code will be provided upon request. All other data generated or analyzed during this study are included in this published article and its supplementary information files. All relevant information about materials used in the study is provided in the Methods section of the text.

## Abbreviations

FACS: Fluorescence activated cell sorting
GO: Gene ontology
Myo: Myoepithelial
PPI: Protein–protein interaction
scRNA-seq: Single-cell RNA-sequencing
UMAP: Uniform manifold approximation projection
