# Supplementary figures and images for: Defining mammary basal cell transcriptional states using single-cell RNA-sequencing
Source: Sci Rep. 2022 Mar 22;12:4893. doi: 10.1038/s41598-022-08870-1 (PMC8940936; doi:10.1038/s41598-022-08870-1)

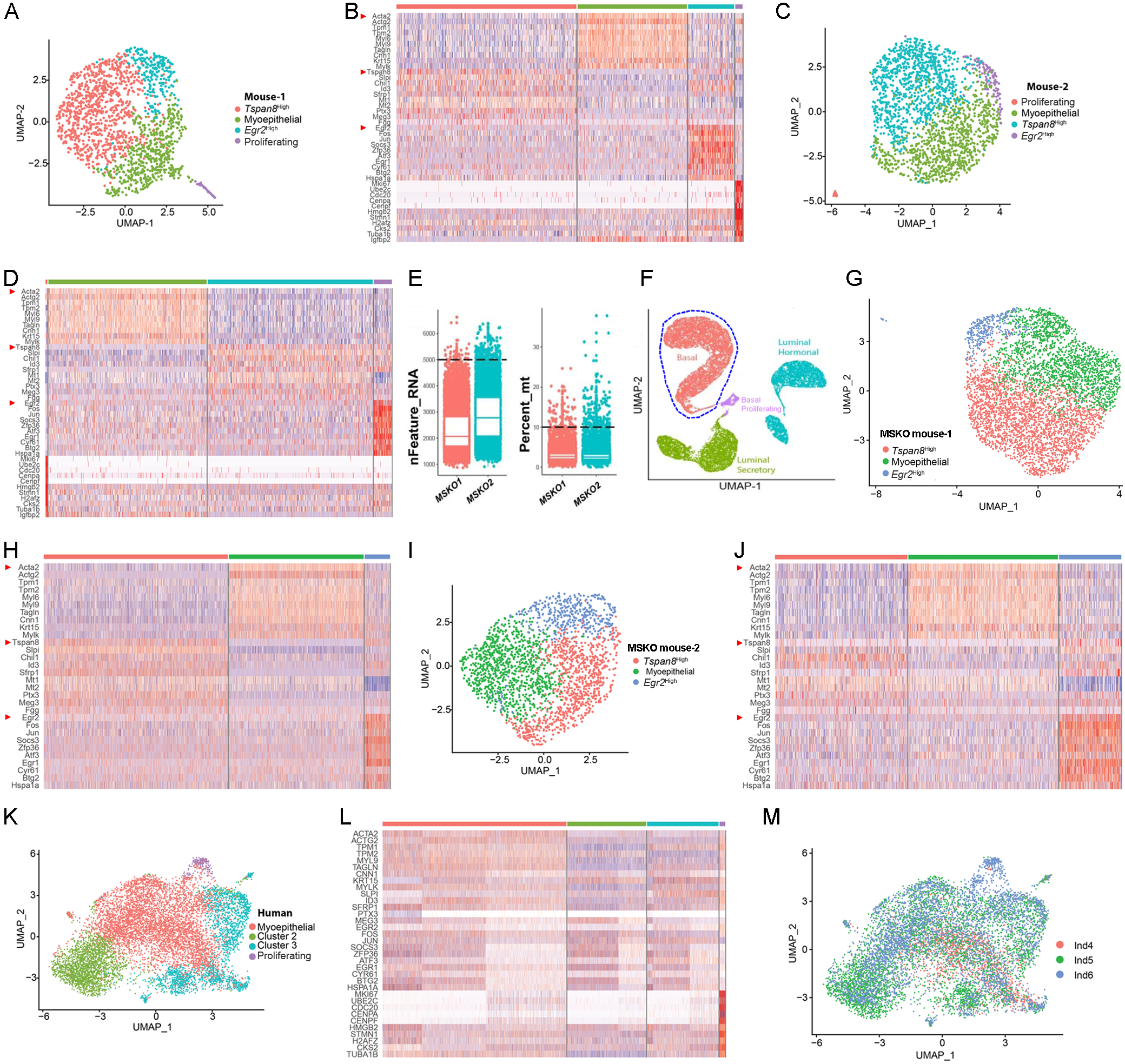

Supplement: Supplementary file 1 — Supplementary Figure S1. [file 41598_2022_8870_MOESM1_ESM.tif]

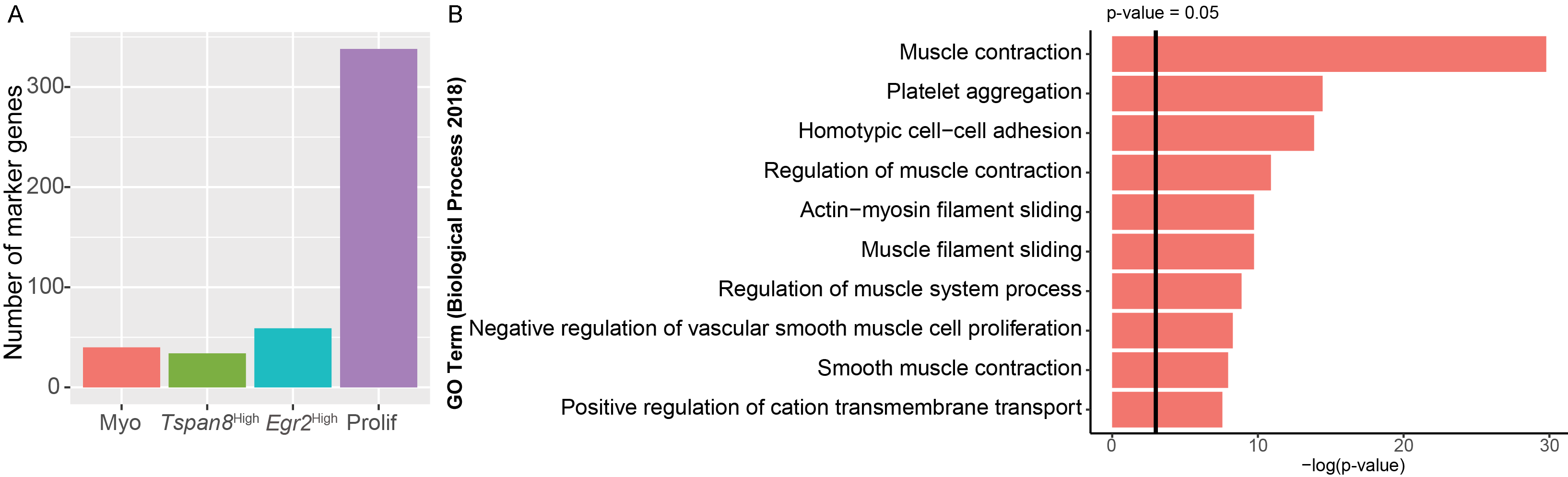

Supplement: Supplementary file 2 — Supplementary Figure S2. [file 41598_2022_8870_MOESM2_ESM.tif]

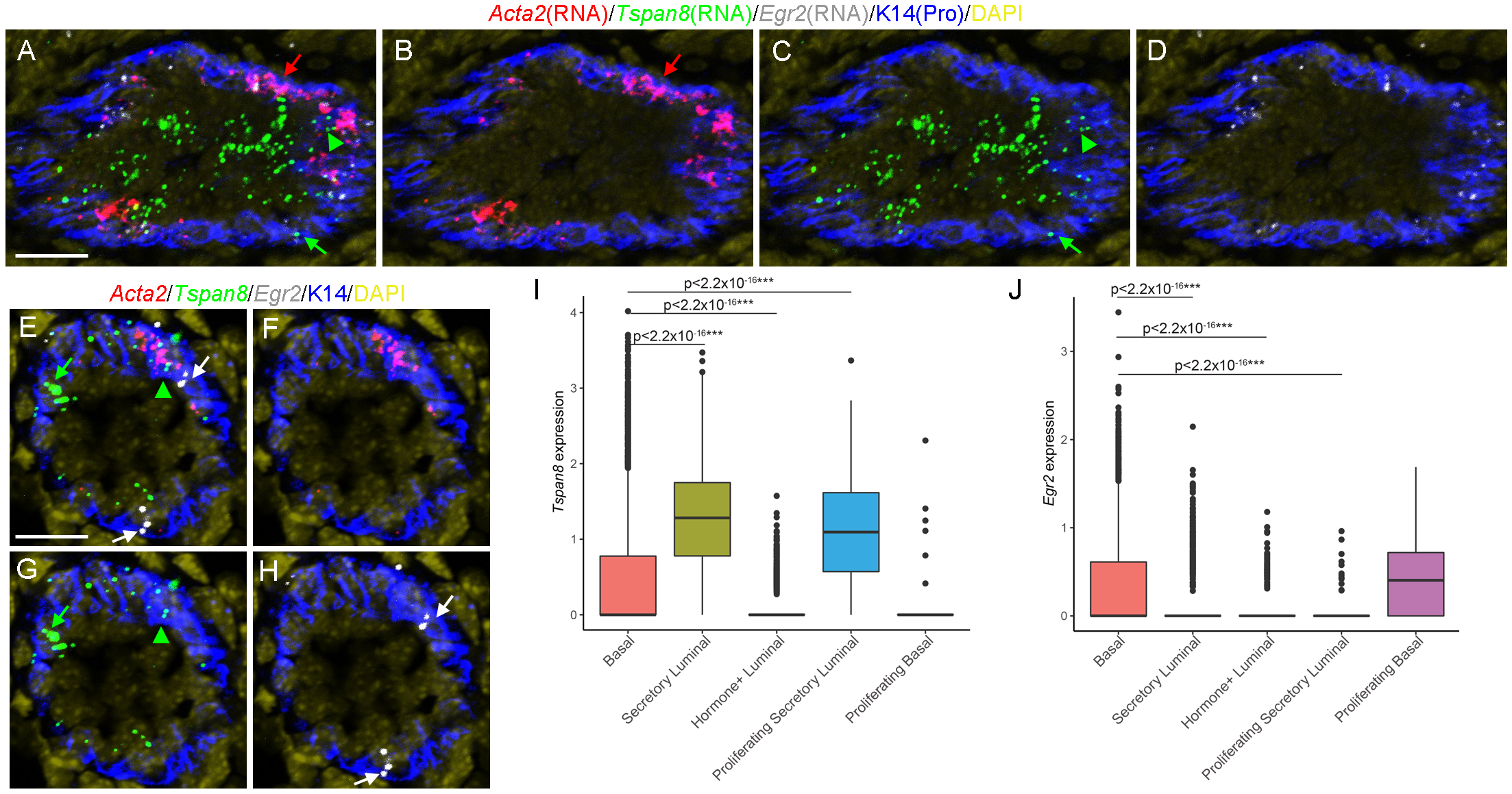

Supplement: Supplementary file 3 — Supplementary Figure S3. [file 41598_2022_8870_MOESM3_ESM.tif]

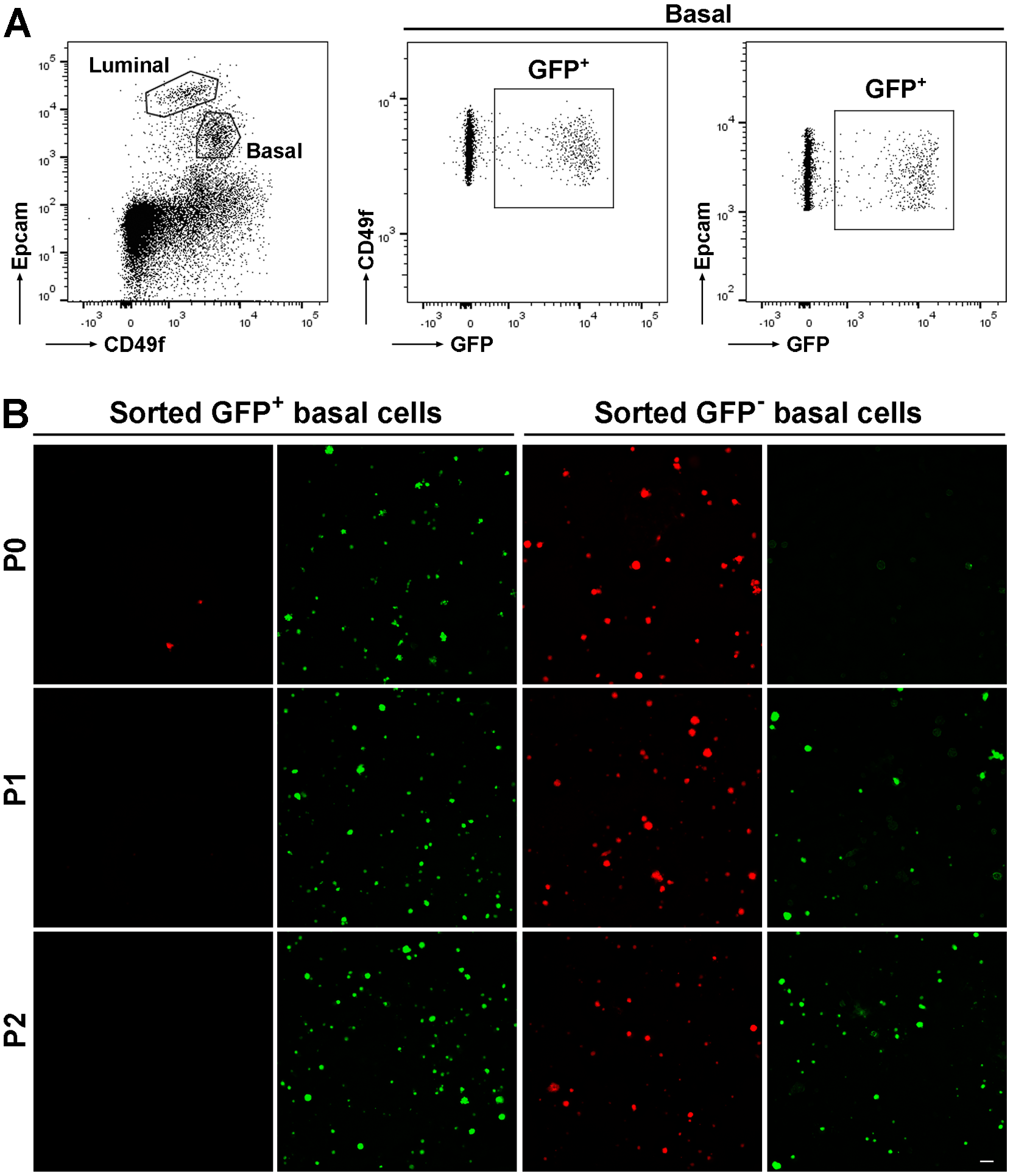

Supplement: Supplementary file 4 — Supplementary Figure S4. [file 41598_2022_8870_MOESM4_ESM.tif]
