# Supplementary material for: Defining mammary basal cell transcriptional states using single-cell RNA-sequencing
Source: Sci Rep. 2022 Mar 22;12:4893. doi: 10.1038/s41598-022-08870-1 (PMC8940936; doi:10.1038/s41598-022-08870-1)
Supplement: Supplementary file 8 — Supplementary Legends. [file 41598_2022_8870_MOESM8_ESM.docx]

**SUPPLEMENTAL TABLES**

**Supplemental Table 1**. List of marker genes for each basal transcriptional state.

**Supplemental Table 2**. Percent of mouse and human basal cells in which the top ten marker genes for the four major mouse basal transcriptional states were detected.

**Supplemental Table** 3. List of genes used for gene scoring analysis with relevant references.

**SUPPLEMENTAL FIGURE LEGENDS**

**Figure S1. Basal cell heterogeneity in mouse and human mammary glands. Related to Figure 1.** (A, C) UMAP projection of basal transcriptional states in wild-type mouse 1 (A) and mouse 2 (C). (B, D) Heatmaps of marker genes of basal transcriptional states (as displayed in Figure 1C) in mouse 1 (B) and mouse 2 (D). (E) Violin plots of the number of genes and total mitochondrial DNA (%) detected per mammary basal cells in mammary epithelial-specific *Zeb1* knockout (MSKO) mice. (F) UMAP projection of all mammary epithelial cells from MSKO mice after quality control filtration in (E). Note that only the “non-proliferating” cells (indicated by a dashed outline) were included in further analysis. (G, I) UMAP projections of basal transcriptional states in the first (G) and second (I) *Zeb1* knockout mouse. (H, J) Heatmaps of marker genes of basal cell states in the first (H) and second (J) *Zeb1* knockout mouse. (K) UMAP projection of transcriptional heterogeneity of *KRT14^+^* cells in the three human individuals and heatmap (L) of the marker genes. (M) UMAP projection of human basal cell heterogeneity colored by individual.

**Figure S2. Number of marker genes and GO terms. Related to Figure 2.**

(A) Bar plots measuring the number of marker genes for each basal transcriptional state. (B) Bar plots of top 10 most significant GO terms identified in the GO Biological Process 2018 library using the marker genes for the “myoepithelial” transcriptional state.

**Figure S3. Additional data on basal transcriptional state marker expression. Related to Figure 3.** (A-H) Additional representative 5-channel (A, E) and 3-channel (B-D, F-H) RNAScope images showing the expression of *Acta2* (red), *Tspan8* (green), *Egr2* (white) in the mammary gland of 10-week-old virgin females. K14 protein immunostaining (blue in all images) marks the basal cells. DAPI stains the nuclei (artificially colored yellow in all images). Red, green, and white arrows indicate a basal cell with only *Acta2*, *Tspan8,* and *Egr2* expression, respectively. Green arrowhead indicates a *Tspan8-*expressing K14-positive cell that resides in basal-luminal border. Scale bar = 20 μm. (I-J) Boxplots showing *Tspan8* (I) or *Egr2* (J) expression in mammary epithelial cell clusters detected by scRNA-seq analysis. *p* values were generated using a Mann-Whitney *U* test.

**Figure S4. Further characterization of GFP^+^ and GFP^-^ basal cells. Related to Figure 5.** (A) Flow profiles to show similar surface marker expression in GFP^+^ and GFP^-^ basal cells. (B) Images of GFP and tdTomato fluorescence in organoids produced by FACS-sorted GFP^+^ and GFP^-^ basal cells from mammary gland of 3-week-old *Egr2-Cre;ROSA26^mTmG^* mice. P0, initial plating. P1 and P2, passages 1 and 2, respectively. Scale bar = 200 μm.
